# Women and gambling-related harm: a narrative literature review and implications for research, policy, and practice

**DOI:** 10.1186/s12954-019-0284-8

**Published:** 2019-03-04

**Authors:** Simone McCarthy, Samantha L. Thomas, Maria E. Bellringer, Rebecca Cassidy

**Affiliations:** 1 Centre for Population Health Research, School of Health and Social Development, Faculty of Health, Deakin University, Geelong, Australia; 2 Gambling and Addictions Research Centre, School of Public Health and Psychosocial Studies, Faculty of Health and Environmental Sciences, Auckland University of Technology, Auckland, New Zealand; 3 Department of Anthropology, Goldsmiths, University of London, London, UK

**Keywords:** Gambling, Women, Gender, Harm, Problem gambling, Public health

## Abstract

**Background:**

While the prevalence of women’s participation in gambling is steadily increasing, there is a well-recognised male bias in gambling research and policy. Few papers have sought to synthesise the literature relating to women and gambling-related harm and provide practical suggestions to guide future research, policy, and practice which take into account the specific nuances associated with women’s gambling.

**Methods:**

A narrative literature review was conducted to review the evidence base on women’s gambling behaviours and experiences of harm. Drawing from strategies used effectively in other areas of public health, key elements for a gendered approach to harm prevention were identified and adapted into practical public health research, policy and practice strategies.

**Results:**

Results indicated a lack of research that explores women’s gambling. Few studies have examined the impact of gambling on the lives of women, with limited understanding of the factors that influence women’s engagement with gambling products, and the impact of industry tactics. A gendered approach was identified as a strategy used successfully in other areas of public health to shift the focus onto women and to ensure they are considered in research. In tobacco control, increasing trends in women’s smoking behaviour were combatted with targeted research, policy and practical initiatives. These key elements were adapted to create a conceptual framework for reducing and preventing gambling harm in women. The framework provides regulatory direction and a research agenda to minimise gambling-related harm for women both in Australia and internationally. Evidence-based policies should be implemented to focus on the influence of gender and associated factors to address gambling-related harm. Practical interventions must take into account how women conceptualise and respond to gambling risk in order to develop specific harm prevention programs which respond to their needs.

**Conclusion:**

A gendered approach to gambling harm prevention shifts the focus onto the unique factors associated with women’s gambling and specific ways to prevent harm. As seen in other areas of public health, such a framework enables harm measures, policies, and interventions to be developed that are salient to girls and women’s lives, experiences and circumstances.

**Electronic supplementary material:**

The online version of this article (10.1186/s12954-019-0284-8) contains supplementary material, which is available to authorized users.

## Introduction

Gambling is a well-recognised public health issue that causes significant harms for individuals, their families and communities [1–3]. Gambling products are more accessible and available than ever before, are embedded within community and online environments, and have become increasingly normalised through their alignment with valued social and cultural activities, such as sport [4]. While research shows that women have similar gambling participation rates as men [5–8], compared with the past when men were more likely to gamble than women [9, 10], there is a clear and well-recognised male bias in the gambling literature [11–13]. This may have had major implications for gambling policy and harm prevention initiatives, particularly if public health initiatives and behavioural treatment models are based on the findings of research conducted predominantly with men.

While there may be a number of reasons for a focus on men in gambling research—including that some gambling products are predominantly used by men [13] and that men are considered to be the most at risk group for problem gambling [6, 8, 14]—recent prevalence studies show similarities between men and women’s overall levels of gambling-related harm and that rates of female problem gamblers are increasing more quickly than males [6]. For example, the Victorian Prevalence Study in Australia (2015) reported that 12.06% of the female adult population were at risk of experiencing harm from their gambling, with significant increases in low-risk gamblers who were assumed to replace non-problem gamblers [6]. Further, new gambling products and environments may increasingly appeal to women and may influence their participation in gambling, their conceptualisation of the risks and benefits of gambling and their experiences of gambling-related harm. It is therefore critical to develop effective evidence-based public health harm prevention strategies which are tailored to the needs of women.

This paper provides a narrative review of current research evidence related to women’s gambling attitudes and behaviours. An additional file also provides a summary of the current evidence base relating to women's gambling and the key findings, methodologies, limitations, and declarations of funding for each research study (Additional file 1). Based on public health approaches in tobacco control which highlighted the importance and effectiveness of gendered approaches to smoking prevention, the paper proposes a framework for a comprehensive gendered approach to gambling research, policy and practice.

### Women and gambling: a narrative review of the evidence base

#### Women’s participation in and harm from gambling

In the last decade, some prevalence studies have demonstrated that gambling participation rates are roughly similar for both women and men [5, 7, 8]. For example, data from the New Zealand National Gambling Study (2012) showed that 80.3% of women and 80.4% of men had gambled in the past year [5], with the British Gambling Prevalence Survey (2010) finding that 71% of women had participated in at least one gambling activity in the past year compared to 75% of men [8]. Similarly, in Canada, a 2009 survey in Quebec found that 68.1% of women and 73.1% of men reported past year gambling participation [15]. In Nordic countries, the gaps between female and male gambling are slightly larger, with a 2011 prevalence study in Finland demonstrating that 73% of women and 83% of men had gambled in the past year [16] and the 2009 Swedish Longitudinal Gambling Study reporting 68.5% of women and 74.7% of men having gambled in the past year [17]. A few studies have also demonstrated increases in the number of women engaging in regular gambling [6–8]. For example, the British Gambling Prevalence Survey documented an increase in the percentage of women gambling at least once a week from 37% in 2007 to 40% in 2010 [8, 18]. This study also showed that younger women, in particular, had the greatest increases in frequency of gambling, across a range of gambling products [8, 19].

With a rise in women’s gambling participation, research has documented increases in the number of women experiencing harm from gambling [6]. In Australia, the Victorian Prevalence Study reported that over one in ten (12.06%) of the Victorian female adult population were at risk of experiencing harm from their gambling, which has gradually increased over time [6]. Other countries report slightly lower gambling risk rates for women, which may be a result of variances in the availability of gambling products, gambling opportunities and cultural differences [8, 14, 16]. There are also significant issues with the way gambling harm is measured in prevalence studies, with researchers identifying that screening tools underestimate the true extent of the harm individuals experience from gambling [20, 21].

There has been very limited qualitative commentary on why regular participation in gambling may be increasing for women. The most common explanation is the increasing ‘feminisation’ of some forms of gambling, with gambling becoming more socially acceptable, safe and less stigmatising for women [6, 22]. This concept, originally mentioned in the Australian Productivity Commission Report [1999], describes a trend of more women gambling, developing problems with gambling and seeking help for gambling following an increase in access and availability of electronic gambling machines (EGMs) [23]. The introduction of casinos, clubs, and hotels that contained EGMs provided glamourous entertainment venues that were attractive to women [24, 25]. This was viewed as a clever marketing tactic that, along with widespread advertising campaigns, promoted gambling as an acceptable leisure activity for women [24–26].

Research in the UK has discussed the ‘re-feminisation’ of gambling, whereby women’s gambling participation decreased following the introduction of betting shops [19]. With gambling predominantly conducted in public and visible spaces, this may have disproportionally discouraged women’s attendance and engagement with products, with a sense among women that they would be judged by other women for their gambling behaviour [19]. However, a shift in the gambling landscape in the UK, which saw an increase in online gambling, an increase in gambling advertising and sponsorships and limited government regulation, legitimatised gambling as a valid recreational activity among women [19]. As gambling products become more prevalent and attitudes towards gambling products become more normalised, we are likely to continue to see this increasing trend of women’s gambling participation.

#### Women’s engagement with gambling products and venues

Researchers have argued that there are a number of assumptions in the gambling literature about women’s gambling behaviour and their engagement with different gambling products, including that women gamble less than men, start gambling at a later age than men and have different product preferences to men [27]. For example, research has suggested that women prefer chance-based forms of gambling, such as lotteries, bingo and EGMs, compared to men who prefer skill-based forms of gambling, such as wagering and poker [6, 7, 18, 28]. Some researchers have argued that this is because women generally avoid products that involve a skill element, suggesting that a lack of technical knowledge about specific games may influence their product preferences [29]. Other researchers have argued that gender roles and expectations, including the activities that boys and girls engage in from childhood [28, 30, 31], may partly protect women from engaging in and experiencing harm from gambling as adults [28, 31]. However, these explanations assume that gender roles and expectations, and therefore activity preferences, are relatively fixed and unchangeable. They do not reflect the current gambling landscape in which a range of products, industry promotional strategies and gambling environments may increasingly expose, appeal to or target women with a range of different gambling opportunities. For example, recent research found that regardless of gender, young people who were engaged in sport had high-level recall of gambling advertising and positive attitudes towards gambling products [32]. This indicates that young women may be equally at risk of gambling harm as young men, when exposed to industry advertising [32].

Recent research has identified changing patterns in women’s gambling, with younger women’s product preferences shifting and/or diversifying towards skill-based forms of gambling [27, 33]. For example, prevalence studies in both Australia and the UK have demonstrated a significant increase in women betting on horse racing [6, 8]. Further, there is some evidence that women’s product preferences are expanding, with women gambling on a range of gambling products [19, 33]. McCarthy and colleagues (2018) found that while, overall, women gambled on EGMs more than any other gambling product, young women in particular gambled on a range of products, including betting on sports and horse racing [33]. This contradicts previous research that has asserted that women have limited interest in skill-based products [29]. Researchers have hypothesised that engagement in gambling and product preferences are ‘socially determined’, and therefore, these changes are more likely to be due to increased exposure to industry marketing and the influence of socio-cultural and environmental factors [19, 27, 34].

#### The role of technology and the changing gambling environment

Advances in technology and the liberalisation of gambling regulation has meant that gambling is more accessible and available than ever before. Researchers have argued that this accessibility has led to an increase in the number of women participating in gambling [35], with some suggesting that newer online platforms remove the some of the stigma associated with women attending male-dominated gambling venues [27]. However, this hypothesis does not explain women’s increased attendance and participation in gambling at physical venues, such as casinos, clubs and hotels [28, 36], as well as the increased popularity of gambling focused events such as horse racing carnivals. While very limited research has investigated why physical or online environments may be increasingly normalised for women, some suggest that the gambling industry may be employing specific strategies to ensure that gambling environments are increasingly attractive, socially acceptable, and inclusive environments for women [37, 38].

#### Gambling marketing and promotions

There is some evidence that the gambling industry may be seeking to appeal to women through marketing; however, the evidence in the academic literature is sparse. Some research into the ‘feminisation’ of gambling has attributed a rise in women’s gambling participation to advertising that is increasingly targeted towards women [19, 39, 40]. For example, researchers have suggested that gambling industries’ tailored marketing to women, alongside an increase in gambling opportunities, has caused gambling to be perceived as an accepted form of entertainment among women [19, 40]. Similarly, research from Sweden reported a “huge amount of aggressive marketing that is exploiting gender stereotypes” (p. 159) that may be influencing women’s gambling behaviour [39].

While much of this marketing remains undocumented in academic research, there is some evidence in the literature of gambling companies using glamour to appeal to women and using female celebrities to promote their products [41]. In the past, women were portrayed in gambling advertising as sexually provocative to appeal to men [42]. However, more recently, wagering companies have used a range of tactics which may appeal to women, including featuring women in lead roles in their promotions [43] and using female celebrities to promote gambling brands on social media sites such as Instagram [44]. There may be parallels with appeal strategies used by the tobacco industry where cigarette companies featured women extensively in advertising campaigns and used slogans that emphasised luxury and elegance [45–49]. However, to date, very limited research has explored the impact of marketing on women’s gambling attitudes and behaviours.

#### The interplay between psychological and social factors

Research has highlighted the range of contextual factors that exist beyond women’s product choices that may influence gambling behaviour [27, 34]. This includes studies that show that particularly for older women, boredom and loneliness are significant motivating factors for gambling, although the same has not been found for men [36, 50], with some women using gambling as a coping mechanism to deal with anxiety and stress [29, 36, 50]. Other studies have explored the role of peers in influencing and motivating women’s gambling behaviour. Australian research has found that young women in particular engage with gambling with their friends as part of their social rituals, suggesting that there may be a degree of socio-cultural acceptance associated with gambling for some groups of young women [33].

While gender is an important predictor of gambling behaviour and trajectory of gambling problems, the effect of gender overlaps with the effect of other psychosocial correlates [51]. Known as the gender-as-proxy hypothesis, researchers suggest that while gender uniquely contributes to gambling patterns, gendered explanations often fail to specify the underlying mechanisms for these differences [51–53]. For example, researchers have argued that while studies show men to be more at risk of problem gambling, this increased risk is not due to their genetic make-up but more accurately relates to an individual’s demographic, economic and health-related factors [54, 55]. Therefore, exploring the psychosocial factors that are associated with gender are more constructive to understanding gambling behaviour in order to develop effective preventative and treatment approaches [51–53]. This warrants greater emphasis on the unique characteristics of at-risk populations, such as older women and indigenous women who may be increasingly vulnerable to gambling-related harm.

#### The health and social impacts of gambling-related harm

Gambling-related harm is also linked to a range of comorbidities for women that are significantly higher than for men [54, 56]. Compared to men, women who experience problem gambling are more likely than men to report comorbidities with anxiety and depression [57, 58], higher frequency of personality disorders [59], co-occurring alcohol-related problems [54, 60], greater psychological distress [61, 62] and are more likely to have experienced childhood abuse [57]. These studies indicate that women’s experiences of gambling-related harm are often part of a complex integration of other issues, whereby harmful gambling behaviour may act as a coping mechanism to mitigate other mental health problems [56].

Few studies have specifically explored women’s lived experiences of harm and how gambling may impact the lives of women. Some research has highlighted the variation of women’s experiences with gambling and how different subgroups of women may conceptualise the harms associated with gambling [37, 63, 64]. It has been recognised that women from ethnic minorities and indigenous communities may be particularly vulnerable to experiencing gambling harm [65]. For example, Hagen et al. (2013) discussed the role in which aboriginal women’s experiences of trauma contributed to their experiences of gambling harm, citing an *“irresistible pull”* towards gambling as a way to escape and cope with problems (p. 366) [63]. Similarly, Māori and Pacific women in New Zealand have been found to disproportionately experience harm from gambling, compared with European women [66, 67]. Research from Morrison (2014) suggests that Māori women experience significant negative effects from EGM gambling; however, these women often only describe the positives of gambling, such as the increased social connections that are not often available to Māori women [66]. Studies with Southeast Asian women in Australia communities have explored their attraction to gambling as a way to relieve stressors in relation to the acculturation process [29, 64, 65]. These studies have found that while gambling environments are social, inclusive and accessible to those of non-English speaking backgrounds, gambling behaviour has been found to significantly negatively impact several aspects of their lives [29, 64, 65].

Positive perceptions about the social and financial benefits of gambling may also impact on how some sub-groups of women may conceptualise and experience gambling-related harm and may be a key risk factor for problem gambling [37, 68, 69]. For example, Thomas and Lewis [37] found older women had lower perceptions of the harm associated with gambling at EGM venues because they felt that there was a trade-off between the social benefits of the gambling environment and the money they lost on EGMs [37]. Similarly, research has demonstrated that older women felt safe and welcomed in gambling environments, reporting that the benefits of attending gambling venues heavily outweighed monetary losses from EGMs [68]. While they described regularly gambling more money on EGMs than they intended to, they perceived gambling as one of the few available and accessible leisure activities for older women [68].

Women may also experience harm due to other people’s gambling. For example, research has demonstrated that partners and family members of gamblers are often impacted by the harms associated with gambling, despite not gambling themselves [70–72]. This includes financial impacts, such as loss of savings and theft, psychological stresses, such as anxiety and depression, and relationship difficulties, such as loss of communication and trust in their partner [70, 73, 74]. Studies also show that gambling problems can become a source of arguments between partners and cause an increase of violence in the relationship [14, 75, 76]. There is a distinct relationship between problem gambling and family violence [70, 72, 77, 78]. Suomi and colleagues [70] reported that family members were often victims of violence by a problem gambler family member, citing an association between gambling behaviour and the violence. Further research indicates that this violence can be bi-directional, with family members of problem gamblers reporting being both victims and perpetrators of family violence [70, 72, 78, 79].

While women were once perceived to be protected from the harms of gambling, a changing gendered landscape, which includes new products, new media, and new technology, means that more women are gambling and experiencing harm from gambling than ever before. Despite this, the lack of gender-specific research has contributed to a perception that gambling problems for women are indistinguishable from those of men, masking the concerns and issues relevant for women who gamble. The following section describes how a gendered approach has been applied in other areas of public health, to guide public health research and harm prevention initiatives.

### Developing a gendered approach to gambling harm prevention

#### Lessons from tobacco control

One of the key problems with understanding the range of factors that may contribute to women’s unique experiences with gambling is the fact that most research relating to women’s gambling is predominantly presented in comparison to the gambling behaviours of men [12, 13]. We argue that a gendered approach, which focuses on the health of men and women separately, is essential in future gambling research, policy and practice in order to develop specific and effective health improvement strategies to meet the needs of different population subgroups [80]. With specific reference to women, researchers in other areas of public health, and in particular in tobacco control, have clearly demonstrated the importance of developing research and policy initiatives which reflect and seek to understand the experiences of women [81, 82]. These include standalone investigations which exclusively examine and report evidence relating to women, including exploring the external factors that influence health outcomes for women and how such outcomes impact their quality of life [81, 82]. For example, the World Health Organization (2007) argued for the importance of incorporating gender into tobacco control measures after extensive research found gendered differences in how tobacco affected individuals and that tobacco control measures were impacted by gender-specific issues, which in turn affected the application and impact of policy interventions [83, 84]. Epidemiological surveys reported an increase in women’s initiation and uptake of smoking, demonstrating the need to focus more attention on women’s smoking behaviours, the factors that influenced their tobacco use and how these factors affected women differently to men [81, 82]. Researchers then used this information to suggest changes to tobacco policies which were guided by a gender framework [82]. This challenged the gender bias that was inherent in tobacco control policies and programs, caused by a lack of gender-specific research and inadequate provision of services relevant for women [82].

Critical to the development of successful tobacco control interventions for women was a robust independent research evidence base, which specifically explored the biological, social, cultural, commercial and economic factors that influenced health outcomes for women [81, 82]. This meant that practical strategies aimed at reducing the harms associated with tobacco use were grounded in an evidence-based understanding of women’s smoking patterns, including the factors that influenced their behaviour [81]. Understanding the range of socio-cultural factors and industry tactics that shape women’s attitudes and beliefs about harmful behaviours were critical to developing smoking cessation and social marketing campaigns targeted specifically towards women [83, 85]. Greaves (2015) stated that:

“The investigations of meanings of smoking are critically important in this shift, as they illustrate the lived experiences and interpretations of women who smoke. These experiences give rise to understanding how to intervene, and what properties of cigarette smoking may have to be replaced in a smoke free life” (p. 1459) [86].

Using a gendered approach, the World Health Organization in 2007 proposed a series of recommendations to incorporate gender into tobacco control measures. For example, the gender and tobacco control policy report recommended collecting and analysing gender-specific information on tobacco use, evaluating the effectiveness of tobacco control measures and using gendered education and communication approaches to increase public awareness and support for enforcement of effective tobacco control policies [83]. Similarly, Samet and Yoon (2010) developed a gender equality framework for tobacco control which aimed to guide policy makers to keep gender in mind when implementing tobacco control measures [82]. Further, Greaves and Jategaonkar (2006) proposed an Ethical Framework for Tobacco Policy, which focused on developing gender sensitive and tailored tobacco programs and policies [84]. This model emphasised collaboration with communities which were potentially more vulnerable to smoking initiation and tobacco use, engaging girls and women in the research and policy processes to better meet their needs [84]. This was done by using collaborative methods to develop authentic partnerships with groups most affected. [84].

Perhaps most importantly, recommendations and frameworks all highlighted that tobacco control harm prevention and reduction initiatives needed to be iterative and flexible and to address the broad context of women’s lives. For example, research evidence provided detailed and nuanced information for clinicians about the range of factors that made smoking cessation difficult for different population subgroups of women [87], enabling programs to be tailored to meet the needs of women from culturally and linguistically diverse communities [88, 89], social support interventions to be developed for women from low socio-economic groups [90–92], a reduction in the stigma associated with seeking support and the provision of, and access to, services for women who were experiencing social and economic disadvantage [92, 93]. Researchers also considered differences in the effectiveness of public education campaigns for women. For example, studies recommended targeting different educational messages to women from different socio-economic groups, rather than relying on messages aimed at the general population which only influenced the behaviours of some groups of women [94–96].

How then may we draw upon the successful approach utilised by public health practitioners, researchers and policy makers in tobacco control, to develop a gendered approach to the prevention and reduction of gambling-related harm?

### A proposed framework for a gendered approach to gambling research, policy and practice

**Fig. 1:**
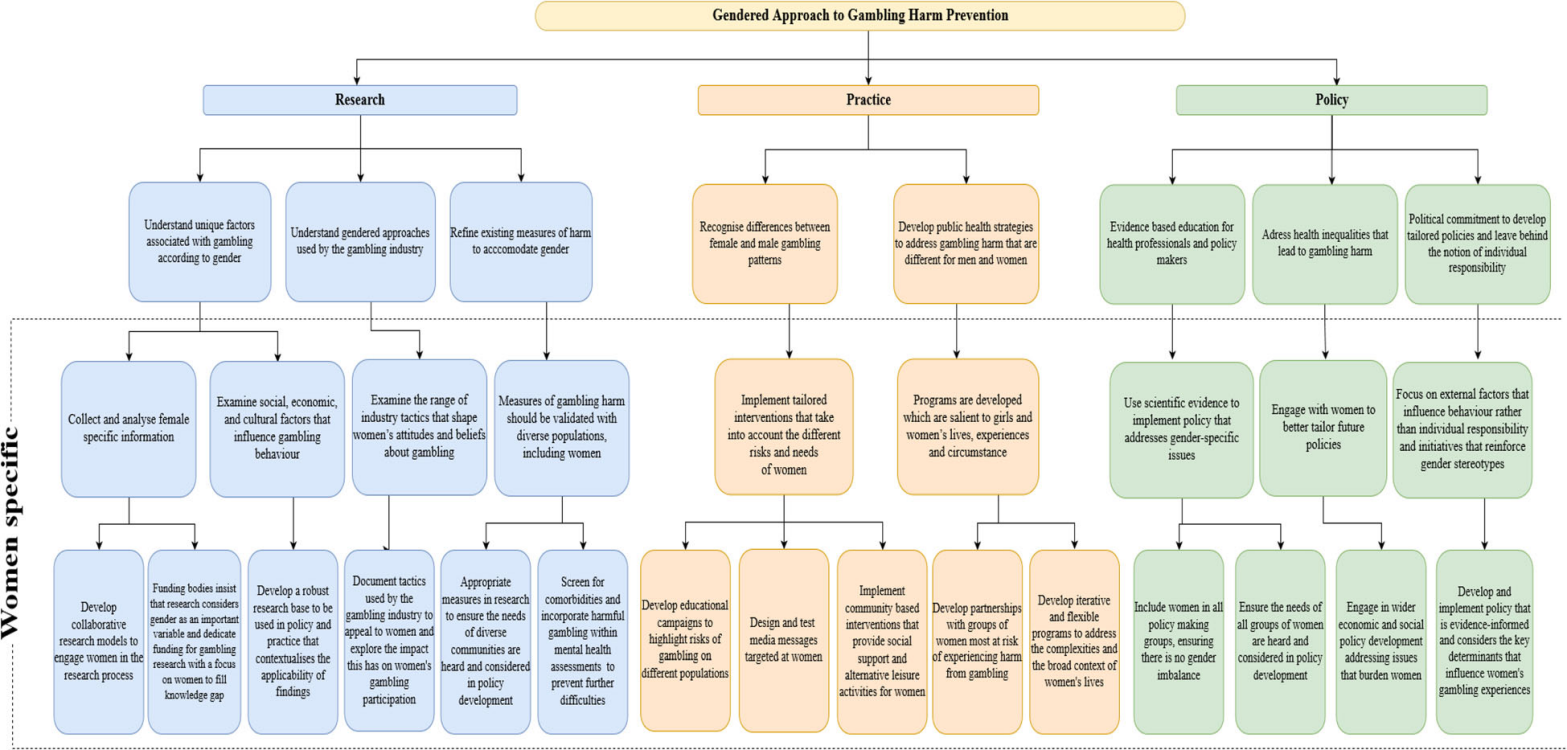
Gendered approach to gambling harm prevention. This framework is designed for preventing and reducing harm from gambling among women and explores and outlines the need for gender diversity in gambling research. The framework provides comprehensive regulatory direction, strategies for practical initiatives and a research agenda to minimise gambling harm for women both in Australia and internationally

The following framework (Fig. 1) has been developed using key findings, recommendations and effective strategies from tobacco control research and policy and has been adapted to a gambling context to specifically address gambling-related harm from a gendered perspective. This framework details specific considerations for research, practice, and policy and outlines practical strategies to identify and address the unique factors associated with women’s gambling in order to prevent and reduce harm.

The framework is divided into three sections; research, practice and policy. Each section is intended to complement and support the other sections.

#### Research

Research shows that women have different experiences with gambling than men. It is therefore important that efforts are made to examine this in detail through a gendered perspective. Similar to tobacco control approaches, this will involve gambling researchers recognising gambling as a public health issue that uniquely affects men and women, considering the social context and broader conditions of women’s lives that may influence gambling behaviour and experiences of harm [83, 84, 97], and being inclusive of the distinct needs and challenges of sub-groups of women [98, 99]. Further, research should also explore the range of individual, socio-cultural, environmental and industry tactics that may contribute to, and influence experiences of, gambling harm [81]. This is essential to understanding the factors that influence women’s wellbeing, their gambling behaviour and the development and maintenance of gambling problems [54]. Collecting and analysing gender-specific information will lead to the development of a robust research base, which will be informative for the development of interventions and policies that prevent and reduce gambling harm experienced by women [81, 82, 98].

In recognition of the rising rates of gambling-related harm among women, funding bodies could accelerate the rate of growth of knowledge about women’s gambling by, for instance, dedicating a proportion of their budget to research which focuses specifically on women. They might also seek to advise all applicants seeking support that every research project will be expected to consider gender and whether or not it is an important variable in the research design of a particular project. The development of a strong evidence base will contribute to the development of effective regulations and interventions that could reduce and prevent gambling harm [81, 100]. Existing measures of gambling harm should be refined based on evidence that takes into account women’s different experiences of gambling harm [82, 84]. This will enable the development of effective measures of gambling harm that specifically capture and respond to women’s experiences and ensure that the needs of diverse communities that are affected by gambling harm are heard and considered in policy development [82].

#### Practice

Effective public health responses to tobacco were informed by the lived experiences of women, which helped to establish precisely “what properties of cigarette smoking may have to be replaced in a smoke free life” (p. 1459) [86]. A similar approach was utilised in other public health harm reduction initiatives to ensure that women and men benefited equally from prevention and recovery programs [81, 98]. We contend that the same approach could be used to prevent and reduce gambling-related harm within different population subgroups, including women. Specifically, evidence about women’s experiences with gambling and the diverse factors that shape their behaviour could inform the development of prevention and treatment strategies that recognise gender differences and ensure that the concerns and experiences of both men and women are an integral part of program development, implementation, and evaluation [98]. Further, a deeper understanding of women's expriences of gambling could contribute to education or mass media campaigns that are targeted to different subgroups of women, are about specific gambling products, and have tailored messages about the risks of these products [54, 86]. It is also important to test messaging strategies and their effectiveness across different population subgroups of women. In tobacco control, targeted messaging was effective in counteracting gendered marketing from harmful industries; on the basis of this parallel evidence an investigation into the effectiveness of a similar approach to reducing gambling-related harm is warranted [82, 83, 96]. For example, a media campaign aimed specifically at women could be designed and tested with input from women who have experienced gambling-related harm.

It is fundamental that research should guide the development of tailored programs that address women’s vulnerabilities to gambling-related harm and consider women’s wellbeing and support networks, as well as economic and health-related factors [84, 101]. For example, women who are vulnerable to participating in high-risk forms of gambling may need community-based interventions that provide alternative leisure activities or safe alternative venues. Evaluation of such gender-sensitive interventions will also be vital to improving outcomes in women’s health [84, 101]. Screening for mental health problems and a history of trauma will also be important to holistically treat women impacted by gambling. This is because gambling-related harm is rarely experienced in isolation and it is likely that women impacted by gambling-related harm also experience mental health comorbidities that may also need to be addressed concurrently for treatment to be effective [54, 56, 57]. Conversely, in the long term, it would be useful if mental health services screened for gambling behaviour and efforts were made to educate mental health professionals about harmful gambling, as this would enable a form of early intervention that could help to prevent the development of problematic gambling or assist women to access support at an earlier stage [56]. Most importantly, community consultation in the design and planning of public health approaches to gambling-related harm, including an indication of whether or not gender is a significant variable, would help to ensure that interventions are useful to those who are most vulnerable.

#### Policy

Tobacco control has demonstrated that a policy approach that focuses on women and men separately enables policy makers to consider gender-specific issues and thus develop regulations that effectively reduce and prevent harm among different populations [82, 83, 102]. In the gambling context, which has not yet reached the same level of attention, research is required to provide evidence for policy makers and public health practitioners to implement policies that address gender-specific issues and to consider the needs of subgroups of the population. This will include understanding how factors external to individual behaviours, such as socio-economic inequality or the impact of product design or marketing tactics, may impact women’s gambling behaviours, testing interventions among smaller groups and then regulating appropriately to protect women. Although the evidence base is currently limited, we contend that every new policy intended to reduce and prevent gambling-related harm should be designed and assessed in relation to gender. To aid in achieving this goal, it would be advisable that all policy-making groups include an equal number of men and women, thus reflecting the gender balance of the communities that they are set up to serve.

## Conclusion

Applying a gendered framework to gambling harm prevention approaches will enable measures, policies and interventions to be developed that are salient to girls’ and women’s lives, experiences and circumstances. The effectiveness of this approach has been clearly demonstrated in other areas of public health including tobacco. Based on these lessons, in order to develop a comprehensive approach to harm prevention and to better protect women and subgroups of women from gambling harm, gender-specific factors must be routinely and consistently considered at research, practice and policy levels.

## Additional file

Additional file 1: Summary of the literature: Women’s gambling. (PDF 250 kb)

## Abbreviation

EGM: Electronic gambling machine
