# Supplementary material for: Women and gambling-related harm: a narrative literature review and implications for research, policy, and practice
Source: Harm Reduct J. 2019 Mar 4;16:18. doi: 10.1186/s12954-019-0284-8 (PMC6399932; doi:10.1186/s12954-019-0284-8)
Supplement: Supplementary file 1 — Summary of the literature: Women’s gambling. (PDF 250 kb) [file 12954_2019_284_MOESM1_ESM.pdf]

Summary of the literature: Women's gambling

#	Authors (year)	Title	Study Methodology	Population studied	Key Findings & implications	Limitations	Funding source
Comparison of gambling behaviour across gender							
1.	Hraba & Lee 1996 [1]	Gender, gambling and problem gambling.	Telephone survey.	Comparison of 1011 men and women (54.6% women) in Iowa (USA).	Women gambled on less products, however there was no gender differences in frequency of gambling, gambling expenditure and time spent gambling. Women were at equal risk of becoming problem gamblers as men.<br><br>The study called for prevention and treatment efforts to be different for men and women.	Results limited by small sample sizes.<br><br>Telephone surveys excluded individuals without telephones and who did not speak English.	Not declared.
2.	Ohtsuka et al. 1997 [2]	Sex differences in pathological gambling using gaming machines.	Survey.	Comparison of 44 men and 60 women in Melbourne recruited from gambling venues (AUSTRALIA).	Identified a shift in the traditional gambling trends with more women becoming addicted to gambling and women equally as susceptible to pathological gambling as men. Hypothesised a growing acceptance of women gambling in society.<br><br>Recommended tailored interventions for women and encourage women to seek help.	The study was limited by the small, convenient sample, which had a higher proportion of men.<br><br>Participants were recruited from EGM venues and only looked at one form of gambling and therefore may not be generalisable to wider populations.	Not declared.
3.	Crisp et al. 2000 [3]	Sex differences in the treatment needs and outcomes of problem gamblers.	Analysis of registration data from clients of Break Even gambling counselling services.	Comparison of 826 men and 694 women who sought help for their own gambling (AUSTRALIA).	Men were more likely to indicate behaviours associated with criminality, relationship breakdown, and job loss. BreakEven gambling counselling services were more likely to be	Results may have occurred due to different counselling processes between genders or other services clients were	The Victorian Government Community Support Fund through the Department of

					deemed successful for women than men.<br><br>Recommended modifying other treatment services to be gender specific due to significant differences between men and women.	attending.<br><br>High dropout rates before treatment was complete may have impacted the evaluation of the gambling counselling services.	Human Services.
4.	Delfabbro 2000 [4]	Gender differences in Australian gambling: A critical summary of sociological and psychological research	Critical summary of gambling literature.	Review of the sociological and psychological research. No inclusion/exclusion criteria.	Women gamble on fewer gambling products than men and have different motivations for gambling.<br><br>To enhance research, the author suggests results for women should always be compared to men.	Author recognises limitations in previous research that limit the ability to draw conclusions.	Not declared.
5.	Hing & Breen 2001 [5]	Profiling Lady Luck: An empirical study of gambling and problem gambling amongst female club members.	Telephone survey.	Comparison of 1743 men and 1257 women who were members of a club in Sydney (AUSTRALIA).	Men and women differed in terms of gambling product preferences and frequency. Similar problem gambling rates.<br><br>Highlighted differences between men and women who gambled and that this may have policy implications. Provided foundation for further research about why differences exist.	Telephone surveys excluded individuals without telephones and who do not speak English.<br><br>Study only looked at Club members from six Clubs in Sydney and thus results may not be generalisable to other regions in Australia.	Not declared.
6.	Grant & Kim 2002 [6]	Gender differences in pathological gamblers seeking medication treatment.	In-depth qualitative interviews.	Comparison of 53 men and 78 women who met DSM-IV criteria for Pathological Gambling Disorder (USA).	Men started gambling at an earlier age compared to women. Women who started gambling at an older age progressed to pathological gambling more quickly than men. Gendered product preference; women preferred EGMs and	Sample included only those in medication treatment and does not reflect the larger problem gambling population. Eligibility criteria may have also limited the	Grant from the National Centre for Responsible Gaming.

					bingo. Both groups were equally likely to seek treatment.<br><br>The study argued for earlier, more aggressive intervention for women's rapid progression problem gambling.	study as those with other psychological disorders or substance abuse histories were excluded from participating.	
7.	Ibáñez et al. 2003 [7]	Gender differences in pathological gambling.	In-depth qualitative interviews and self-report questionnaires.	Comparison of 47 men and 22 women seeking treatment for pathological gambling (SPAIN).	Gendered differences in product preferences, however similar pathological gambling severity. High levels of comorbidity with other disorders, with men displaying high rates of alcohol abuse and antisocial personality disorder and women reporting a history of physical abuse.<br><br>The need for gender differences and comorbidity of pathological gambling to be incorporated in treatment.	Participants were recruited from an outpatient treatment program and therefore excluded pathological gamblers who were not seeking help.<br><br>The questionnaires were self-reported which may have influenced findings.	Grant FIS 99/0011-01 from the Spanish Ministry of Health.
8.	Volberg 2003 [8]	Has there been a" feminization" of gambling and problem gambling in the United States?	Telephone surveys.	Comparison of 5024 men and women in four state-wide surveys in the US that represented the general population (USA).	Women were less likely to gamble regularly than men, however rates of problem gambling were similar. Gender, social class and ethnicity were associated with product preference. Increased gambling and problem gambling among women coincided with the introduction of EGMs. Recommend implementing measures to prevent gambling problems for different subgroups, including women.	Study does not specify gender breakdown of sample.<br><br>Each survey used different screening tools to measure problem gambling, limiting comparability between surveys used and the overall validity of the study's findings.	The study was funded by the National Institutes of Health, Maryland.

9.	McMillen et al. 2004 [9]	Help-seeking by problem gamblers, friends and families: A focus on gender and cultural groups.	In-depth qualitative interviews.	Comparison of 16 men and women who have sought or considered seeking help for gambling problems for themselves or someone else. Sample consisted of 9 problem gamblers (4 women and 5 men) and 7 family members (6 women and 1 man) (AUSTRALIA).	Highlighted that features of the gambling environment contribute to gambling problems including the location to clubs and convenient access to gambling. Cultural and gender differences influenced variations in gambling behaviour and impact that are not being addressed through support services.<br><br>Recommended the development of effective strategies and a wider range of problem gambling support services.	Participants were self-identified problem gamblers which limits external validity.<br><br>The sample was skewed towards a middle-class population<br><br>The authors acknowledge that methods of recruitment could also have been more culturally sensitive.	The ACT gambling and Racing Commission (GRC).
10.	Blanco et al. 2006 [10]	Sex differences in subclinical and DSM-IV pathological gambling: results from the National Epidemiologic Survey on Alcohol and Related Conditions.	Analysis of data from the National Epidemiologic Survey on Alcohol and Related Conditions survey.	Comparison of men and women in a nationally represented sample of 43,093 adults (USA).	Men gambled more frequently than women and were more likely to be pathological gamblers. Women developed the disorder later in life and were more likely than men to gamble to relieve a depressed mood.<br><br>Highlighted gender differences for tailored intervention.	Study does not specify gender breakdown of sample.<br><br>Secondary data analysis limits understanding of the characteristics and speed of transition from subclinical and DSM-IV pathological gambling.	National Institute on Alcohol Abuse and Alcoholism.
11.	Heater & Patton 2006 [11]	Gender differences in problem gambling behaviour from help-line callers	Analysis of data collected from calls to a problem gambling help-line.	Comparisons of 176 men and 441 women who called a problem gambling help-line (CANADA).	Women called the help line more often than men however were more likely to call because they were concerned about a partner's gambling. More women than previous years called for concern over their own gambling.<br><br>Implications for more research, treatment and prevention	Data was drawn only from a help-seeking population and may not represent all those experiencing harm from gambling.<br><br>Social desirability and interpretation biases in the way that participants	Manitoba Lotteries Corporation.

					programs targeted at women who gamble and a need to improve help seeking among men.	interpreted and responded to questions.	
12.	Potenza, Maciejewski & Mazure 2006 [12]	A gender-based examination of past-year recreational gamblers.	Secondary analysis of data from the 1998 Gambling Impact Behaviour Study.	Comparison of 1131 men and 1231 women over the age of 18 (USA).	While men engaged in heavier recreational gambling compared to women. Both men and women had similar motivations to gamble and experienced similar levels of impact on mental health and general functioning. The study found an association between alcohol use and recreational gambling for both men and women.<br><br>Highlighted the need for research to consider gender-related influences on recreational and problematic gambling.	Data collection method meant that causal effects could not be determined and measures used were not validated.<br><br>Self-report and gender-based bias as women are more likely to report health problems than men.	Supported in part by: National Institute on Drug Abuse grants and Women's Health Research at Yale.
13.	Ellenbogen, Derevensky & Gupta 2007 [13]	Gender differences among adolescents with gambling-related problems.	Analysis of data from five self-report surveys.	Comparison of 2,750 boys and 2,563 and girls between the ages of 12 and 18 (CANADA).	In contrast to adult studies, adolescent boys and girls experiencing gambling problems reported similar risk factors and consequences. Boys were more likely to report gambling problems and endorse physiological and psychological symptoms<br><br>Argued for similar treatment strategies for adolescent boys and girls.	Measures were different across 5 studies, limiting the comparability of the studies.<br><br>Studies also used different population groups, including schools that spoke different languages which may have affected the validity of results.	Grant from Social Science and Humanities Research Council, Fonds de recherche Santé, Ministère de la Santé et des Services sociaux, and Loto-Quebec.
14.	Crisp et. al 2004 [14]	Not the same: A comparison of female and male clients	Analysis of registration and assessment data from	Comparison of 826 men and 694 women who sought help for their own	Found women were almost just as likely to attend BreakEven counselling services as men	Self-reporting methods may have led to inaccurate data. The data	The Victorian Government Community

		seeking treatment from problem gambling counselling services	clients of BreakEven gambling counselling services.	gambling (AUSTRALIA).	despite a perception that problem gamblers were exclusively men. Hypothesised that the increase in women using services was due to the feminisation of gambling and that this service was appealing to women as it was held in discrete community health centres.<br><br>Suggests that other treatment services that are effective for men may not be effective for women.	set also contained some incomplete data.<br><br>Findings were not representative of those who do not seek help and therefore limited understanding of how services can appeal to this population.	Support Fund through the Department of Human Services.
15.	Toneatto & Wang 2009 [15]	Community treatment for problem gambling: sex differences in outcome and process.	Evaluation of cognitive behavioural treatment of men and women seeking treatment for problem gambling.	Comparison of 44 men and 16 women who were receiving outpatient cognitive behavioural treatment for problem gambling (CANADA).	Women who were problem gamblers were more likely than men to be also experiencing mental health issues. Men reported more positive treatment outcomes and reduced severity of their gambling problems than women. Men rated treatment components of CBT to be more helpful than women.<br><br>CBT may be insufficient in addressing women-specific treatment needs.	This study had a small sample size, particularly of women. This limits the generalisability and validity of the results.<br><br>Self-reported data was not verified with clinical data which the authors state limit the internal validity of the study.	Not declared.
16.	Stevens & Young 2010 [16]	Who plays what? Participation profiles in chance versus skill-based gambling.	Analysis of the Northern Territory gambling prevalence survey (2005).	Comparison of 1,172 men and women (47.6% women) from the Northern Territory who had gambled in the previous 12 months (AUSTRALIA).	Skill-based gambling was associated with men and occurred in male-dominated gambling environments, such as racetracks. However, chance based gambling was found not found to be associated with women.	Telephone surveys limit individuals without a landline telephone.<br><br>Sample did not reflect the population of the northern territory. For example the proportion of Indigenous	The Community Benefit Fund of the Northern Territory Government, Australia.

					Recommended further investigation of the social contexts of gambling, such as the increased availability of gambling products.	Australians were under-represented.	
17.	Svensson et al. 2011 [17]	Gendered gambling domains and changes in Sweden.	Telephone interviews.	Comparison of men and women who were regular gamblers in 1997/98 (sample of 6,674) and 2008/09 (sample of 15,000) (SWEDEN).	Men's gambling patterns have changed over time, with men gambling more on chance games than 10 years ago. Women's gambling preferences have not changed, but saw an increase in women gambling on horses after the gaming industry targeted marketing at women. No obvious signs of a feminisation of gambling as men gamble more frequently and have more problems with gambling than women.<br><br>Further exploration of gambling environments as places contributing to the differences in gambling behaviour between men and women.	Study does not specify gender breakdown of sample.<br><br>Difficulties in comparing two studies from different time periods. This included the use of different measures contributing to inconsistencies in analysis. This led to some participant data being excluded.<br><br>The gambling environment has changed over time with online gambling not prevalent in the initial study and thus was not examined in the comparison study.	Funded by the Swedish National Institute of Public Health.
18.	Donati, Chiesi & Primi 2013 [18]	A model to explain at-risk/problem gambling among male and female adolescents: Gender similarities and differences.	Survey.	Comparison of 994 boys and girls (36% girls) attending high school students in Italy with different levels of gambling involvement (ITALY).	Adolescent boys' gambling was associated with perceived economic profit from gambling and peer gambling behaviours. Parental gambling behaviours predicted gambling in adolescent girls. Gambling was seen as a less peer-approved activity for girls,	Findings were based on self-reported data therefore may not accurately reflect gambling behaviour.<br><br>Limited generalisability to other populations	Not declared.

					and a more socially desirable activity for boys.<br><br>Study identified a need to develop prevention programs that educate young people problem gambling.	outside Italian high school students.	
19.	Hing et al. 2014 [19]	A comparative study of men and women gamblers in Victoria.	Secondary analysis of 2009 Victorian gambling prevalence survey.	Comparison of 5508 men and 5727 women who gamble in Victoria (AUSTRALIA).	Men experienced gambling problems at a higher rate than Women. Men preferred skill-based forms of gambling and women preferred chance-based gambling forms. EGMs provided women an escape from stress, loneliness and boredom.<br><br>Men and EGM players should be the primary targets for public health interventions.	Used a non-validated version of the PGSI to measure problem gambling severity which limits comparability to other studies.<br><br>Not all respondents were asked the same questions and sample size varied across the analyses.	The Victorian Responsible Gambling Foundation through the Grants for Gambling Research Program.
20.	McCormack, Shorter & Griffiths 2014 [20]	An empirical study of gender differences in online gambling.	Online survey.	Comparisons of 800 men and 175 women who gamble online from all over the world, predominately from the UK and USA, recruited from international online gambling websites.	Online gamblers were more likely to be men and those at risk of gambling problems were also more likely to be men. Gendered differences in reasons for gambling online with women gambling to practice for free, spend less money and to cure boredom. Women had increased feelings of guilt and shame compared to men.<br><br>Implications for developing tailored interventions due to gendered differences in gambling motivations and online gambling experiences.	The sample was not representative of any population, and only 18.4% of the sample were women. This may have affected the results and their generalisability.<br><br>The retrospective self-reporting method may provide inaccurate responses and is subject to recall bias.	Not declared.

21.	Svensson & Romild 2014 [21]	Problem gambling features and gendered gambling domains amongst regular gamblers in a Swedish population-based study.	Telephone interviews.	Comparison of 2048 men and 1143 women who were regular gamblers subsampled from the Swedish longitudinal gambling study (SWEDEN).	Men preferred skill-based forms of gambling in a public setting, and women preferred chance-based gambling and refrain from gambling in masculine domains. Women who gambled regularly were just as likely as men to be problem gamblers, but when controlling for age, women were more at risk.<br><br>Separate analyses for men and women are important in order to unmask gender inequality and differences.	Quantitative methodologies did not allow for clarity about motives behind gambling behaviour.<br><br>Effect sizes for some sections of the analysis were small.<br><br>The sample was over represented for young people, people born outside of Sweden and individuals on welfare.	The Swedish National Institute of Public Health.
22.	Wardle 2015 [PhD Thesis] [22]	Female gambling behaviour: a case study of realist description.	Secondary data analysis of large-scale government surveys: the Health Survey for England, the British Gambling Prevalence Survey series and the Taking Part survey.	Comparison of men and women from four government surveys that represent the general population in the UK (UK).	Found that in the UK there has been a process of re-feminisation of gambling. This is due to changes in regulatory frameworks and gambling environments, actions of the gambling industry in targeting women, and increased availability of gambling.<br><br>The thesis highlighted the changing nature of women's gambling behaviour and the need to develop theories that represent the complexities of how women's gambling behaviour is shaped.	Study does not specify gender breakdown of sample.<br><br>Limited by the methodologies used in the secondary data sources.<br><br>The use of quantitative measures meant findings were limited to outcomes and did not explore the contextual influences than may have led to these outcomes.	Not declared.
23.	Baxter et. al 2016 [23]	Gender differences in felt stigma and barriers to help-seeking for problem gambling	Focus groups using concept mapping and brainstorming activities.	Comparison of 10 men and 18 women who gamble, family members of gamblers and health care providers who	Shame and stigma perceived as detrimental to help-seeking by men and women. Men felt shame in admitting emotional vulnerability and women felt	Small sample which was recruited from a single gambling venue in an urban setting, limiting	Ontario Problem Gambling Research Centre.

				deliver gambling treatment (CANADA).	shame admitting having an addiction and being enticed by gambling venues.<br><br>Highlighted the need to consider gendered perceptions of what is viewed as stigmatizing and barriers to help-seeking.	generalisability to others experiencing harm.<br><br>Sample had an older mean age which may have influenced the results.	
24.	Haw & Holdsworth 2016 [24]	Gender differences in the temporal sequencing of problem gambling and other disorders.	Survey.	Comparison of 144 men and 123 women who were problem gamblers in treatment aged between 18 and 82 years (AUSTRALIA).	Problem gamblers were found to have comorbidities with other disorders, such as depression and substance use disorders. Women developed problems from gambling after experiencing other disorders whereas men started developing gambling problems before experiencing other disorders. Average age of onset for gambling problems was much later for women than men.<br><br>Strategies should be put in place to prevent women with other disorders from developing gambling problems.	The use of problem gamblers who were in treatment limits the generalisability of the findings.<br><br>Survey was self-reported and relied heavily on past behaviours which may have affected the results.<br><br>Did not show the relationship between problem gambling and other disorders limiting internal validity.	Gambling Research Australia.<br><br>The authors have received funding support and provided consultancies to organisations directly and indirectly benefiting from gambling.
25.	Hing et al. 2016 [25]	Risk factors for gambling problems: An analysis by gender.	Secondary analysis of the 2009 Victorian gambling prevalence survey.	Based on the same material as study [19], comparison of 5508 men and 5727 women who gamble in Victoria (AUSTRALIA).	Men were more likely to be at risk of experiencing harm compared to women. Gambling on EGMs was a risk factor for both genders, but risk was heightened for women. Other risk factors including young adult age, not speaking English and unemployment increased risk for both gendered.	Used a non-validated version of the PGSI to measure problem gambling severity which limits comparability to other studies.<br><br>Not all respondents were asked the same questions	The Victorian Responsible Gambling Foundation.<br><br>All authors have received funding support and provided

					Identification of distinctive risk factors by gender may prompt more focus on the public health of women in relation to gambling.	and sample size varied across the analyses.	consultancies to organisations that directly or indirectly benefit from gambling.
26.	Kim et al. 2016 [26]	Gender difference among helpline callers: Prospective study of gambling and psychosocial outcomes.	Telephone interviews with individuals who called the national problem gambling helpline	Comparison of 64 men and 86 women who called a gambling helpline in regards to their own gambling (New Zealand).	Women who called compared to men who called reported higher problem gambling severity whereas men had a longer history of gambling problems and were more than twice as likely to access treatment. Both men and women reported significant improvement from treatment.<br><br>More needs to be done to encourage women to access treatment and call helplines earlier.	Small sample size and the lack of control group limits the ability for the authors to draw accurate conclusions.	The New Zealand Ministry of Health.
27.	Edgren et al. 2017 [27]	Gender comparison of online and land-based gamblers from a nationally representative sample: Does gambling online pose elevated risk?	Analysis of the cross-sectional Finnish Gambling 2015 survey.	Comparison of 3555 men and women (46.2% women) above the age of 18 who had gambled in the previous 12 months (FINLAND).	Online gamblers were more likely than land-based gamblers to be younger, familiar with computer gaming and engaged in multiple forms of gambling, regardless of gender. For women, online gambling related to higher relative expenditure and at-risk and problem gambling.<br><br>The rise of online gambling may lead to an increase in women experiencing gambling harm. Potential for gendered tailored interventions.	Potential bias and inaccurate responses due to self-report method.<br><br>Overlap between those who gambled online and those who gambled at venues may have affected the results.<br><br>Small effect sizes of age groups.	Ministry of Social Affairs and Health (the 52 Appropriation of the Lotteries Act)

28.	Khanbhai, Smith and Battersby 2017 [28]	Gender by preferred gambling activity in treatment seeking problem gamblers: A comparison of subgroup characteristics and treatment outcomes.	Analysis of data from the Statewide Gambling Therapy Service. This including clients' gambling severity screens, measures of psychological distress and alcohol use measures, as well as reports from clinicians regarding each client's treatment.	Comparison of 166 men and 169 women who have sought treatment for problem gambling (AUSTRALIA).	Women were found to be older, have lower alcohol misuse and have greater psychological distress. No differences in treatment outcomes between genders.<br><br>Found Cognitive Behavioural Therapy (CBT) to be effective across diverse populations and aids in addressing underlying comorbidities.	Self-report scales used may also lead to underreporting of gambling behaviours and comorbidities.<br><br>Sample limited to help-seeking populations and therefore CBT may not be effective for all population groups.	Conducted through the Flinders Centre for Gambling Research
29.	Castrén et al. 2018 [29]	The relationship between gambling expenditure, socio-demographics, health-related correlates and gambling behaviour—a cross-sectional population-based survey in Finland	Analysis of the cross-sectional Finnish Gambling 2015 survey.	Comparison of 1833 men and 1418 women aged 15-74 who were past year gamblers with gambling expenditure data available (FINLAND).	Gambling expenditure was associated with gender, with men spending more on gambling than women. 28.5% of women's gambling expenditure was from problem and pathological gamblers compared to 20.8% of men's. Low income groups contributed more of their income to gambling.<br><br>Highlighted vulnerable groups and a need for early interventions.	Data analysed was self-reported which may be influenced by participant bias.<br><br>When the sample was divided into age groups, these groups were small and effect sizes were not significant.	The Ministry of Social Affairs and Health, Helsinki, Finland (appropriation under section 52 of the Lotteries Act).
30.	Castrén, Heiskanen & Salonen 2018 [30]	Trends in gambling participation and gambling severity among Finnish men and women: cross-sectional population surveys in 2007, 2010 and 2015	Analysis of Finnish cross-sectional gambling surveys in 2007, 2010 and 2015	Comparison of 6785 and 6936 women aged 15-74 who were randomly sampled from 3 Finnish gambling surveys (FINLAND).	Gambling participation for both men and women increased between 2007 and 2015. Findings suggest a feminisation of gambling with an increase in women's gambling participation, women who have gambling problems and women who seek	Differences between each survey including response rates, time of year data was collected and the description of the survey may limit the comparability between each data set.	The Ministry of Social Affairs and Health, Helsinki, Finland (appropriation under section 52 of the Lotteries Act).

					help. This may be due to the rise in online gambling appealing to women.<br><br>Recommended implementing age and gender specific public health measures that effectively prevent, mitigate and treat gambling related harm.	Self-report methodology may have led to an under reporting of gambling behaviours due to social desirability bias.<br><br>Limitations associated with the measure used to assess gambling related harm	
31.	Delfabbro, Thomas and Armstrong 2018 [31]	Gender differences in the presentation of observable risk indicators of problem gambling	Online survey	Comparison of 580 men and 605 women across two Australian studies exploring gambling behaviour (AUSTRALIA).	Symptoms for problem gambling are similar for both men and women. However, men more likely to report anger and frustration when gambling and women report more strong emotional reactions.<br><br>Implications for staff in venues identifying those with problem gambling characteristics.	Potential for under reported of gambling behaviours due to the use of self-report methodology.<br><br>The convenience sampling may limit the generalisability of the results.	Gambling Research Australia.
32.	Weidberg et al. 2018 [32]	Gender differences among adolescent gamblers.	Survey.	Comparison of 942 boys and 814 girls aged 14-17 from 22 Spanish secondary schools (SPAIN).	Men were more than three times as likely as women to be at-risk or problem gamblers. Men engaged in a greater number of gambling activities, and showed more severe gambling patterns than women. Specific risk factors for gambling by gender were identified.<br><br>Implications for increased gender-sensitive prevention efforts, and stronger regulatory measures to protect adolescents.	Authors acknowledged the limitation in the research design where researchers were unable to control for independent and extraneous variables.	Grant from the Council for Economy and Work and a pre-doctoral grant from the Spanish Ministry of Economy and Competitiveness.

Focus on women who seek help							
33.	Hallebone 1999 [33]	Women and the new gambling culture in Australia.	In-depth qualitative interviews with 10 women.	10 women self-identified as problem gamblers attending problem gambling counselling services. (AUSTRALIA).	Women who were problem gamblers were likely to have previously suffered from violence and abuse and gambled as a way to regain a sense of identity and worth. These women resented the introduction of EGMs in Victoria and their increasing availability.<br><br>Authors suggested that responsible gambling education could help problem gamblers retain control over their gambling	Small sample derived from women seeking help from gambling and does not represent those who may not seek help.	Not declared.
34.	Davis & Avery 2004 [34]	Women who have taken their lives back from compulsive gambling: Results from an online survey.	Online survey.	252 women in recovery for compulsive gambling (USA).	Recovered women reported more negative consequences compared to women still gambling. This included mental health deterioration, relationship breakdowns and illegal activities. Study hypothesised that women still gambling were in denial about the harm they were experiencing and less likely to endorse consequences. High rate of co-occurrence substance abuse and depression, troubled family histories, and severe gambling harm.<br><br>Professional help should be informed with the complexities of problem gambling and the growing problem among women.	The study recruited from online websites and may over represent online gamblers and exclude venue-based gamblers.<br><br>Sample was overrepresented by white women of middle to high class and underrepresented women from different cultural and low income backgrounds.	Not declared.

35.	O’Brien 2005 [Dissertation] [35]	A qualitative study of the development and maintenance of pathological gambling in women: and making the choice to recover.	In-depth qualitative interviews with 8 women.	8 women in recovery from pathological gambling (USA).	Gambling problems progressed rapidly following a difficult emotional event or loss. Gambling provided a sense of social inclusion in an environment that was safe and comfortable. Treatment was found to be effective for women to set boundaries and learn to take responsibility.<br><br>Advocated for women specific treatment as women who are pathological gamblers commonly have a history of maltreatment.	Design of the study excluded pathological gamblers who were not in the process of recovery and therefore does not represent all pathological gamblers.<br><br>Qualitative methods and phenomenological approach limits the generalisability of the results beyond the sample.	Not declared.
36.	Piquette-Tomei et al. 2008 [36]	Group therapy for women problem gamblers: A space of their own.	In-depth interviews.	14 women participating in group counselling for problem gamblers (CANADA).	Women perceived counselling groups were effective for women when they were accessible and in a comfortable and accepting environment where it was safe to share information. The women in the study preferred women-only groups and were empowered when others shared their stories. There were significant barriers to accesses services including feelings of shame and guilt.<br><br>Implications for increased women-only group counselling in safe, accessible and accepting spaces.	The study only explored the effectiveness of group therapy on women who were attending these services and therefore limits understanding of what may be effective to reach women not already attending services.<br><br>The study involves a small sample of women and this may not represent all problem gambling women.	The Alberta Gaming Research Institute.
37.	Dowling, Smith and	The family functioning of female	Surveys.	53 women who were pathological gamblers attending a treatment	Pathological gamblers perceived that family members would not be able to provide support. Partners	Small sample derived from treatment-seeking pathological gamblers.	Not declared.

	Thomas 2009 [37]	pathological gamblers.		program for gambling related problems and 29 of their partners and 40 of their children (AUSTRALIA).	reported elevated rates of relationship dysfunction. Unknown whether women gamble to escape relationship and family problems or that their gambling may cause these issues.<br><br>Treatment services should address relationship issues associated with women's gambling and targeted interventions for family members.	Absence of a control group limits the ability for authors to make definitive conclusions.<br><br>Measures used were unable to determine causality between relationship problems and women's gambling behaviour.	
38.	Piquette & Norman 2012 [38]	An all-female problem-gambling counselling treatment: Perceptions of effectiveness.	In-depth qualitative interviews.	4 women who were in a 12 week women-only gambling treatment group as well as follow-up focus groups (CANADA).	Women in the treatment group found women-only groups to be helpful and stated that they would prefer to use women-only treatment in the future. Participants stated the group provided connectedness and validation, helped build positive relationships, and empowered women to share their own live experiences.<br><br>Made a strong case for treatment services that are supportive, well-facilitated and gender specific to improve problem gambling interventions for women.	Small sample size of women from similar ethnic backgrounds limits the study's generalisability.<br><br>The study included only women who sought treatment to end their gambling behaviour which excluded women who used services to cut back from gambling.	Not declared.
39.	Mestre-Bach et. al 2018 [39]	Sociodemographic and psychopathological predictors of criminal behaviour in women	Face to face clinical interviews.	273 treatment-seeking women diagnosed with gambling disorder. Comparison between 61 women who reported a history of criminal	Women with a gambling disorder who reported committing illegal acts were significantly younger and had more severe gambling related problems than those without a criminal history. These	Authors recognised social desirability bias as data was self-reported and perceive this could have led to underreporting of criminal behaviours.	Grants from Instituto de Salud Carlos III and cofounded by FEDER funds/European

		with gambling disorder.		behaviour and 212 who had no criminal history (SPAIN).	women were also categorised as having high levels of impulsive decision making, possess antisocial tendencies and have higher levels of alcohol abuse.<br><br>Recommended considering women's high levels of impulsivity and dysfunctional personality traits when developing harm reduction interventions.	Sample consisted of women being treated for a diagnosed gambling disorder and may only represent women who seek treatment.	Regional Development Fund (ERDF), a way to build Europe. CIBERobn and CIBERSAM are an initiative of ISCIII
Focus on distinct subgroups of women							
40.	Hong Chui 2008 [40]	True stories: Migrant Vietnamese women with problem gambling in Brisbane, Queensland.	Qualitative interviews.	Two Vietnamese women with gambling problems and their 2 case workers (AUSTRALIA).	Gambling in the Vietnamese community is viewed negatively and is associated with shame. Migrants are vulnerable to depression, loneliness, and isolation due to limited family support which may lead to problem gambling.<br><br>Implications for gambling services to include family in the recovery process and work in partnership with existing community services and resources.	The study explores the experiences of only two women from one migrant community in Australia. Difficult to generalise results beyond this small sample.	Not declared.
41.	Hagen et al. 2013 [41]	A big hole with the wind blowing through it: Aboriginal women's experiences of trauma and problem gambling.	In-depth qualitative interviews.	34 Aboriginal women classified as problem gamblers (CANADA).	Highlights the link between Aboriginal women's experiences of poverty and social trauma and the development of problem gambling. These women described gambling as an escape mechanism which only served to maintain their trauma.	The authors acknowledged that if the study were replicated at a different point in time, different themes may have been observed.	Grant awarded through the Alberta Gaming Research Institute.

					Importance of screening problem gamblers for social trauma, suggesting that gambling behaviour is a coping mechanism for underlying issues. Therefore problem gambling cannot be treated in isolation.	Participants were self-identified as problem gamblers which limits other women who do not recognise harm.	
42.	Pattinson & Parke 2017 [42]	The experience of high-frequency gambling behaviour of older adult women in the United Kingdom: An interpretative phenomenological analysis.	In-depth qualitative interviews.	10 women over the age of 60 who were gambling at least once every 14 days (UK).	Participants gambled to fill voids and emotionally escape from stressful life events. Older women were vulnerable to gambling harm because of negative experiences with aging including loss of social network from retirement, loss of caregiving responsibilities and loss of spouse. Perceived gambling environments as positive and the only available leisure activity for older women with restricted mobility, and that monetary losses were outweighed by the benefits of gambling.<br><br>Identified the need for alternative leisure activities and better support for older women.	Difficult to generalise results beyond small sample.<br><br>Potential for pre-existing biases from researchers that may affect the interpretation of the results.	PhD bursary, awarded from the Responsible Gambling Trust charity.
43.	Riley et al. 2017 [43]	Problem gambling among female prisoners: lifetime prevalence, help-seeking behaviour and association with incarceration.	Surveys.	74 women in prison in South Australia (AUSTRALIA).	Association found between women's problem gambling and criminal offending with a higher proportion of women in prison reporting lifetime prevalence of problem gambling than the general population. Found that women are vulnerable to engaging	Self-report method may have led to inaccurate results due to social desirability bias.<br><br>Small sample and low response rate may not represent the wider	The Department for Correctional Services Community Grant

					in criminal activities when gambling to pay back debts.<br><br>Provides evidence that women in prison are vulnerable to gambling problems. Implications for rehabilitation and treatment programs in prison.	population of women in prison.	
44.	Pfund et al. 2018 [44]	Influence of Social Interaction on Women College Students' Electronic Gambling Machine Behaviour	Randomised control trial	109 women in college who were recreational gamblers (USA).	Social interaction was found to influence gambling behaviour for women in college.<br>Women placed larger bets on EGMs when exposed to warm social interaction compared to cold interaction and control groups. For this group, presence of another person was identified as a risk factor for excessive gambling.<br><br>The influence of the social context must be considered in responsible gambling interventions.	This study only examined gambling behaviour using EGMs and used a sample of non-problem gamblers.<br><br>The controlled environment may not accurately reflect that of a real gambling venue and does not consider other gambling domains such as online.	Not declared.
Focus on women who gamble							
45.	Trevorrow & Moore 1998 [45]	The association between loneliness, social isolation and women's electronic gaming machine gambling.	Survey.	Comparison between 95 gambling and non-gambling women (AUSTRALIA).	Gambling was viewed as a socially approved activity by all women and gambling women were not motivated to gamble by loneliness or isolation. However, women who were classified as problem gamblers were lonelier than the rest of the sample. Hypothesised that lonely women who gamble may be more vulnerable to losing control.	No control group was used and therefore differences between gambling and non-gambling women may not have been due to their gambling behaviour.<br><br>Measures of problem gambling were self-reported.	Not declared.

					Considered loneliness as a risk factor for developing problems with gambling for women.		
46.	Scannell et al. 2000 [46]	Women' coping styles and control over poker machine gambling.	Surveys.	163 women who played poker machine, recruited from gaming venues (AUSTRALIA).	Found a relationship between coping styles and gambling. Women who relied on emotion-focused coping strategies had lower levels of control than women who used problem-focused approaches. Emotion focused strategies may be ineffective to regulate or control ones gambling behaviour and women using this coping mechanism may use gambling as an avoidance strategy. This may contribute to the development of pathological gambling.<br><br>More research is needed to explore individual coping styles and the effect this has on experiencing gambling harm.	This research relied on self-report methodology for several measures of gambling behaviour and coping strategies and therefore results may be inaccurate due to the sensitive nature of gambling and social desirability bias.	Not declared.
47.	Berry, Fraechlich & Toderian 2002 [47]	Women's experiences of gambling and problem gambling.	Phone-In interviews and further in-depth interviews, observations at gambling environments, analysis of information from callers to a problem gambling helpline, file data from a	36 women who called in to a phone service to talk about their gambling and 17 of these women who agreed to participate further, two Winnipeg casinos, 72 women who called a helpline seeking services for problem gambling, 103 case files from the firm of	Women who gamble were found to be a diverse group who gambled for entertainment, to win money and to escape negative aspects of their lives. Identified experiencing financial problems, relationship difficulties and guilt. Women also expressed concern over family members' gambling behaviour. Women suggested improvements to services in	Volunteer bias due to method of women calling the researchers.<br><br>Only a small amount of women in the study had previously sought help from gambling and therefore this limits the authors' ability to make	The Ontario Problem Gambling Research Centre

			bankruptcy trustee and file data on clients presenting with gambling problems at an addiction service.	bankruptcy trustees declared by women, 35 gambling admission files of women at an addiction service (CANADA).	community health centres and providing group counselling.<br><br>Implications for regulations and treatment to consider differences in men and women's gambling and address issues the reflect women's experiences.	recommendations to improving services.	
48.	Li 2007 [48]	Women's ways of gambling and gender-specific research	Participant observation and in-depth interviews	Observations at three casinos on 40 occasions and interviews with 7 women who gamble (CANADA).	Gambling played an important role in women's lives as a response to difficult life situations. Women gambled for many reasons including gambling as hope for a better life, for an escape, as therapy to heal emotional wounds, as a reward, and for socialisation. The gambling culture of women was influenced by an interplay of gender, class, and society.<br><br>Called for future research with a focus on women who gamble and the context of their lives.	Results are not representative of all women as only a small sample of 7 women were interviewed, whom were all of low socioeconomic status.	The Ontario Problem Gambling Research Centre
49.	Corney & Davis 2010 [49]	The attractions and risks of Internet gambling for women: A qualitative study.	In-depth qualitative interviews.	25 women who gambled frequently on the Internet (UK).	Found online gambling to be more attractive than other gambling venues for women. Internet gambling was seen as a fun and social activity, accessible from home, anonymous and private. Women were aware of the risks of online gambling such as the use of not 'real' money and providing an escape from other problems.	Problem gamblers who were recruited were in the process of trying to stop gambling, with participants stating that they would be unable to talk about gambling at the height of their addiction.<br><br>An incentive of a monetary voucher was	The Responsible Gambling Fund in the UK.

					Implications for identifying women engaging in online gambling to reduce their risk of excessive gambling.	given to participate which may have skewed the sample.	
50.	Holdsworth, Nuske & Breen 2012 [50]	Only the lonely: An analysis of women's experiences of poker machine gambling.	In-depth qualitative interviews.	20 women who were EGM players, 10 of whom identified as recreational gamblers and 10 who had sought and received help for their gambling (AUSTRALIA).	Women who were recreational gamblers gambled for enjoyment whereas help-seeking gamblers gambled relieve feelings of loneliness and were emotionally vulnerable as a result of a crisis in their lives. Recreational gamblers were also at risk of developing problems. Women's gambling behaviour attributed to gender role expectations across life stages.<br><br>Prevention strategies to target women at different risk levels including public health education and information to assist women to continue gambling socially.	Participants were self-identified as either a recreational gambler or as having sought help from gambling. This identification was found not to be reflected in the PGSI scores of some women.	The National Association for Gambling Studies.
51.	Holdsworth, Nuske & Breen 2013 [51]	All mixed Up together: Women's experiences of problem gambling, comorbidity and co-occurring complex needs.	In-depth qualitative interviews.	Using the same sample as the previous study [50], the study included 20 women who were EGM players, 10 of whom identified as recreational gamblers and 10 who had sought and received help for their gambling (AUSTRALIA).	Help-seeking gamblers had a range of comorbid issues including mental illnesses, substance abuse, relationship breakdown and financial problems. These women were likely to relapse into problematic gambling behaviour as they struggled through other issues.<br><br>Complex needs and underlying issues need to be identified so that concerns can be holistically addressed.	Participants were self-identified as either a recreational gambler or as having sought help from gambling. This identification was found not to be reflected in the PGSI scores of some women.	Not declared.

52.	Järvinen-Tassopoulos 2016 [52]	Gender in focus - gambling as an individual, social and political problem.	Editorial	Discussion of existing literature with no inclusion/exclusion criteria specified.	Discussed women' gambling as part of their socialisation in the family context, connected to socio-cultural environments. Described gambling and problem gambling as highly gendered activities and suggested regulation and preventative measures that are not gender neutral.<br><br>Advocated for a gendered perspective to provide new insights into gambling practices and its relationship with gender.	This editorial delivers a summary of existing literature however provides little critical analysis.	Not declared.
53.	Nuske, Holdsworth & Breen 2016 [53]	Significant life events and social connectedness in Australian women's gambling experiences.	In-depth qualitative interviews.	20 women who were EGM players (AUSTRALIA).	Women either started gambling as a social activity or as a way to cope after a significant life event. Women with gambling problems described gambling after feeling socially isolated from others and lacked support networks. Recreational gamblers had adequate support and managed their gambling to expected gender role expectations.<br><br>Highlighted the need for social connectedness for women after significant life events to mitigate harm.	Small sample was used that overrepresented older women with a mean age of 51.5. Emergence of themes such as isolation may not be reflective of women who play EGMs but for women in that age group.	The National Association for Gambling Studies (NAGS).
54.	McCarthy et al. 2018 [54]	Women's gambling behaviour, product preferences, and perceptions of product harm:	Online survey	A sample of 509 women aged 16-88 including 324 women who had participated in a gambling	Younger women most likely to report risk of problem gambling than older women Younger women gambled more frequently and across more products than any	The sample was overrepresented by women experiencing harm from gambling compared to the general	The Victorian Responsible Gambling Foundation Competitive

		Differences by age and gambling risk status		activity in the last 12 months (AUSTRALIA).	other age group. These women engaged with gambling products for social reasons suggesting gambling becoming a regular part of young women' lives. The paper suggested that different subgroups of women may conceptualise harm differently.<br><br>Called for future research to investigate women's gambling and the differences between subgroups of women.	population due to the online survey methodology.<br><br>When the sample was divided into age groups, these groups and their effect sizes were small.<br><br>The study only compared use of four gambling products and may not represent women who gamble on other products.	Grants Scheme revenue (*via hypothecated taxes from gambling).*
Literature review of women's gambling							
55.	Mark & Lesieur 1992 [55]	A feminist critique of problem gambling research.	Gendered analysis of gambling literature.	Reviewed the representation of women who were pathological gamblers in psychopathological-diagnostic, physiologically, psychological, sociologically and treatment oriented articles. 43 papers were included in this review.	Gambling literature is dominated by research that over represents men in their sample and studies that fail to discuss gender-related findings. This has had implication on the development of gambling theories.<br><br>Calls for more research with women who gamble, results to be presented by gender and use measures that are validated using samples of both men and women.	Authors critique the current literature and explore the limitations of these studies such as their failure to make gender-related analyses and that there are only five existing studies that address pathological gambling among women.	Not declared.
56.	Martins et al. 2002 [56]	Pathological gambling in women: a review.	Literature review.	Review of the last 10 years of problem gambling studies with special emphasis on clinical and epidemiological aspects of women who gamble.	Men and women have different gambling preferences and attitudes towards gambling, with women progressing to gambling problems at a quicker rate than men. Evidence that problem gambling in women is higher than	The gambling literature on women is sparse and often limited to research that is performed in clinical settings, disregarding women who	FAPESP Brazil (The State of São Paulo Research Funding Agency).

					predicted however, women are underrepresented in treatment services and therefore clinical research.<br><br>Argues the importance of specific strategies for prevention and treatment for women that are evidence-informed.	do not seek help for problem gambling.<br><br>The studies included in the review were not specified in the paper.	
57.	McKay 2005 [57]	Double jeopardy: Older women and problem gambling.	Literature review.	Review of existing literature of older women (55 and over) experiencing problem gambling.	Increases in older women's gambling and problem gambling were associated with the introduction and increased availability of EGMs. Interplay of individual, social factors and game design may make older women vulnerable. Concern that prevalence studies may not accurately reflect the magnitude of women's gambling problems. Future research into different subgroups to inform treatment and prevention strategies.	Limitations of the research used in this literature review are critiqued by the author. This includes that prevalence studies may not accurately reflect the magnitude of women's problem gambling and that most research in this field is quantitative.<br><br>The studies included in the review were not specified in the paper.	Not declared.
58.	Wenzel & Dahl 2009 [58]	Female pathological gamblers - a critical review of the clinical findings.	Critical review of the literature concerning clinical characteristics of women who are pathological gamblers compared to men who are pathological gamblers.	Review of literature from 1970 to 2007 that compared men and women over the age of 18 with a clinical diagnosis of problem or pathological gambling. 28 papers were included in this review.	Women started gambling at an older age than men, and typically had a faster progression to problem gambling. Women gambled on fewer products and preferred games of chance. Women were motivated to gamble to escape from emotional distress.	The reliability of the literature review and comparisons and conclusions made is limited due to methodical issues in the studies reviewed.	No funding

					Implications for tailored interventions with specific focus on emotional needs for women.		
59.	Holdsworth, Hing & Breen 2012 [59]	Exploring women's problem gambling: A review of the literature.	Literature review.	Review of existing literature with no inclusion/exclusion criteria specified.	Reviewed and discussed features of women's gambling suggesting that men and women have different gambling practices including their gambling motivations, product preferences, progression to gambling problems and help seeking behaviour. Attribute differences to gender role theory and social capital theory.<br><br>Gender specific research is important to identify specific life stages when women are at risk of developing gambling problems. This information should be used to implement evidence informed prevention strategies.	The reliability of the literature review is limited by the various limitations of the research studies used.<br><br>Findings may not be relevant due to lack of contemporary gender specific research and therefore explanations may be inaccurate and not reflect women's current gambling culture.<br><br>The studies included in the review were not specified in the paper.	Not declared.

References

1. Hraba J, Lee G. Gender, gambling and problem gambling. Journal of Gambling Studies. 1996;12(1):83-101.
2. Ohtsuka K, Bruton E, DeLuca L, Borg V. Sex differences in pathological gambling using gaming machines. Psychological Reports. 1997;80(3):1051-7.
3. Crisp BR, Thomas SA, Jackson AC, Thomason N, Smith S, Borrell J, et al. Sex differences in the treatment needs and outcomes of problem gamblers. Research on Social Work Practice. 2000;10(2):229-42.
4. Delfabbro P. Gender differences in Australian gambling: A critical summary of sociological and psychological research. Australian Journal of Social Issues. 2000;35(2):145-58.
5. Hing N, Breen H. Profiling lady luck: An empirical study of gambling and problem gambling amongst female club members. Journal of Gambling Studies. 2001;17(1):47-69.
6. Grant J, Kim S. Gender differences in pathological gamblers seeking medication treatment. Comprehensive Psychiatry. 2002;43(1):56-62.
7. Ibáñez A, Blanco C, Moreryra P, Sáiz-Ruiz J. Gender differences in pathological gambling. The Journal of clinical psychiatry. 2003;64(3):295-301.
8. Volberg RA. Has there been a "feminization" of gambling and problem gambling in the United States? Journal of Gambling Issues. 2003;8:1-33.
9. McMillen J, Marshall D, Murphy L, Lorenzen S, Waugh B. Help-seeking by problem gamblers, friends and families: A focus on gender and cultural groups. ACT, Australia: Centre for Gambling Research, Regnet, Australian National University 2004.
10. Blanco C, Hasin DS, Petry N, Stinson FS, Grant BF. Sex differences in subclinical and DSM-IV pathological gambling: results from the National Epidemiologic Survey on Alcohol and Related Conditions. Psychological Medicine. 2006;36(07):943-53.
11. Heater J, Patton D. Gender differences in problem gambling behaviour from help-line callers. Journal of Gambling Issues. 2006(16).
12. Potenza MN, Maciejewski PK, Mazure CM. A gender-based examination of past-year recreational gamblers. Journal of Gambling Studies. 2006;22(1):41-64.
13. Ellenbogen S, Derevensky J, Gupta R. Gender differences among adolescents with gambling-related problems. Journal of Gambling Studies. 2007;23(2):133-43.
14. Crisp BR, Thomas SA, Jackson AC, Smith S, Borrell J, Ho W-y, et al. Not the same: a comparison of female and male clients seeking treatment from problem gambling counselling services. Journal of Gambling Studies. 2004;20(3):283-99.

15. Toneatto T, Wang JJ. Community treatment for problem gambling: sex differences in outcome and process. Community Mental Health Journal. 2009;45(6):468-75.
16. Stevens M, Young M. Who plays what? Participation profiles in chance versus skill-based gambling. Journal of Gambling Studies. 2010;26(1):89-103.
17. Svensson J, Romild U, Nordenmark M, Månsdotter A. Gendered gambling domains and changes in Sweden. International Gambling Studies. 2011;11(2):193-211.
18. Donati MA, Chiesi F, Primi C. A model to explain at-risk/problem gambling among male and female adolescents: Gender similarities and differences. Journal of Adolescence. 2013;36(1):129-37.
19. Hing N, Russell A, Tolchard B, Nower L. A comparative study of men and women gamblers in Victoria. Victoria: Victorian Responsible Gambling Foundation; 2014.
20. McCormack A, Shorter G, Griffiths M. An empirical study of gender differences in online gambling. Journal of Gambling Studies. 2014;30(1):71-88.
21. Svensson J, Romild U. Problem gambling features and gendered gambling domains amongst regular gamblers in a Swedish population-based study. Sex Roles. 2014;70(5-6):240-54.
22. Wardle H. Female gambling behaviour: a case study of realist description [PhD thesis]: University of Glasgow; 2015. Accessed 20 April 2017. Available from: http://theses.gla.ac.uk/6117/.
23. Baxter A, Salmon C, Dufresne K, Carasco-Lee A, Matheson FI. Gender differences in felt stigma and barriers to help-seeking for problem gambling. Addictive Behaviors Reports. 2016;3:1-8.
24. Haw J, Holdsworth L. Gender differences in the temporal sequencing of problem gambling with other disorders. International Journal of Mental Health and Addiction. 2016;14(5):687-99.
25. Hing N, Russell A, Tolchard B, Nower L. Risk factors for gambling problems: An analysis by gender. Journal of Gambling Studies. 2016;32(2):511-34.
26. Kim HS, Hodgins DC, Bellringer M, Abbott M. Gender differences among helpline callers: Prospective study of gambling and psychosocial outcomes. Journal of gambling studies. 2016;32(2):605-23.
27. Edgren R, Castrén S, Alho H, Salonen AH. Gender comparison of online and land-based gamblers from a nationally representative sample: Does gambling online pose elevated risk? Computers in Human Behavior. 2017;72:46-56.

28. Khanbhai Y, Smith D, Battersby M. Gender by preferred gambling activity in treatment seeking problem gamblers: A comparison of subgroup characteristics and treatment outcomes. Journal of gambling studies. 2017;33(1):99-113.
29. Castrén S, Kontto J, Alho H, Salonen AH. The relationship between gambling expenditure, socio-demographics, health-related correlates and gambling behaviour—a cross-sectional population-based survey in Finland. Addiction. 2018;113(1):91-106.
30. Castrén S, Heiskanen M, Salonen AH. Trends in gambling participation and gambling severity among Finnish men and women: cross-sectional population surveys in 2007, 2010 and 2015. BMJ open. 2018;8(8):e022129.
31. Delfabbro P, Thomas A, Armstrong A. Gender differences in the presentation of observable risk indicators of problem gambling. Journal of gambling studies. 2018;34(1):119-32.
32. Weidberg S, González-Roz A, Fernández-Hermida JR, Martínez-Loredo V, Grande-Gonsende A, García-Pérez Á, et al. Gender differences among adolescent gamblers. Personality and Individual Differences. 2018;125:38-43.
33. Hallebone E. Women and the new gambling culture in Australia. Loisir et Société/Society and Leisure. 1999;22(1):101-25.
34. Davis DR, Avery L. Women who have taken their lives back from compulsive gambling: Results from an online survey. Journal of Social Work Practice in the Addictions. 2004;4(1):61-80.
35. O'Brien KL. A qualitative study of the development and maintenance of pathological gambling in females: And making the choice to recover [Doctoral dissertation]: University of Minnesota; 2015. Accessed 22 March 2018. Available from: https://conservancy.umn.edu/bitstream/handle/11299/175280/OBrien_umn_0130E_16002.pdf?sequence=1&isAllowed=y.
36. Piquette-Tomei N, Norman E, Dwyer SC, McCaslin E. Group therapy for women problem gamblers: A space of their own. Journal of Gambling Issues. 2008(22):275-96.
37. Dowling N, Smith D, Thomas T. The family functioning of female pathological gamblers. International Journal of Mental Health and Addiction. 2009;7(1):29-44.
38. Piquette N, Norman E. An all-female problem-gambling counseling treatment: Perceptions of effectiveness. Journal of Groups in Addiction & Recovery. 2013;8(1):51-75.
39. Mestre-Bach G, Steward T, Granero R, Fernández-Aranda F, Talón-Navarro MT, Cuquerella À, et al. Sociodemographic and psychopathological predictors of criminal behavior in women with gambling disorder. Addictive behaviors. 2018;80:124-9.

40. Hong Chui W. True stories: migrant Vietnamese women with problem gambling in Brisbane, Queensland. Journal of Social Work Practice in the Addictions. 2008;8(2):276-80.
41. Hagen B, Kalishuk RG, Currie C, Solowoniuk J, Nixon G. A big hole with the wind blowing through it: Aboriginal women's experiences of trauma and problem gambling. International Gambling Studies. 2013;13(3):356-70.
42. Pattinson J, Parke A. The experience of high-frequency gambling behavior of older adult females in the United Kingdom: An interpretative phenomenological analysis. Journal of Women & Aging. 2017;29(3):243-53.
43. Riley BJ, Larsen A, Battersby M, Harvey P. Problem gambling among female prisoners: lifetime prevalence, help-seeking behaviour and association with incarceration. International Gambling Studies. 2017;17(3):401-11.
44. Pfund RA, Ginley MK, Whelan JP, Peter SC, Wynn BS, Suda MT, et al. Influence of Social Interaction on Women College Students' Electronic Gambling Machine Behaviour. Journal of Gambling Issues. 2018(38).
45. Trevorrow K, Moore S. The association between loneliness, social isolation and women's electronic gaming machine gambling. Journal of Gambling Studies. 1998;14(3):263-84.
46. Scannell ED, Quirk MM, Smith K, Maddern R, Dickerson M. Females' coping styles and control over poker machine gambling. Journal of gambling studies. 2000;16(4):417-32.
47. Berry R, Fraehlich C, Toderian S. Women's experiences of gambling and problem gambling. Winnipeg: Ontario Problem Gambling Research Centre; 2002.
48. Li J. Women's ways of gambling and gender-specific research. Sociological Inquiry. 2007;77(4):626-36.
49. Corney R, Davis J. The attractions and risks of Internet gambling for women: A qualitative study. Journal of Gambling Issues. 2010;24:121-39.
50. Holdsworth L, Nuske E, Breen H. Only the lonely: an analysis of women's experiences of poker machine gambling. Gambling Research: Journal of the National Association for Gambling Studies. 2012;23(2):17.
51. Holdsworth L, Nuske E, Breen H. All mixed Up together: Women's experiences of problem gambling, comorbidity and co-occurring complex needs. International Journal of Mental Health and Addiction. 2013;11(3):315-28.
52. Järvinen-Tassopoulos J. Gender in focus - gambling as an individual, social and political problem. Nordic Studies on Alcohol & Drugs. 2016;33(1):3-6.

53. Nuske EM, Holdsworth L, Breen H. Significant life events and social connectedness in Australian women’s gambling experiences. Nordic Studies on Alcohol and Drugs. 2016;33(1):7-26.
54. McCarthy S, Thomas S, Randle M, Bestman A, Pitt H, Cowlishaw S, et al. Women’s gambling behaviour, product preferences, and perceptions of product harm: Differences by age and gambling risk status. Harm Reduction Journal. 2018;15(22):1-12.
55. Mark ME, Lesieur HR. A feminist critique of problem gambling research. Addiction. 1992;87(4):549-65.
56. Martins SS, Lobo DS, Tavares H, Gentil V. Pathological gambling in women: a review. Revista do hospital das clínicas. 2002;57(5):235-42.
57. McKay C. Double jeopardy: Older women and problem gambling. International Journal of Mental Health & Addiction. 2005;3(2):35-53.
58. Wenzel HG, Dahl AA. Female pathological gamblers - a critical review of the clinical findings. International Journal of Mental Health and Addiction. 2009;7(1):190-202.
59. Holdsworth L, Hing N, Breen H. Exploring women's problem gambling: A review of the literature. International Gambling Studies. 2012;12(2):199-213.
